# "Just like fever": a qualitative study on the impact of antiretroviral provision on the normalisation of HIV in rural Tanzania and its implications for prevention

**DOI:** 10.1186/1472-698X-9-22

**Published:** 2009-09-09

**Authors:** Maria Roura, Alison Wringe, Joanna Busza, Benjamin Nhandi, Doris Mbata, Basia Zaba, Mark Urassa

**Affiliations:** 1 Centre for Population Studies, Department of Epidemiology and Population Health, London School of Hygiene and Tropical Medicine, 49-51 Bedford Square, London, WC 1B 3DP, UK; 2 TAZAMA Project, Tanzanian National Institute for Medical Research (NIMR), PO Box 1462, Mwanza, Tanzania

## Abstract

**Background:**

Once effective therapy for a previously untreatable condition is made available, a normalisation of the disease often occurs. As part of a broader initiative to monitor the implementation of the national antiretroviral therapy (ART) programme, this qualitative study investigated the impact of ART availability on perceptions of HIV in a rural ward of North Tanzania and its implications for prevention.

**Methods:**

A mix of qualitative methods was used including semi-structured interviews with 53 ART clinic clients and service providers. Four group activities were conducted with persons living with HIV. Data were analyzed using the qualitative software package NVIVO-7.

**Results:**

People on ART often reported feeling increasingly comfortable with their status reflecting a certain "normalization" of the disease. This was attributed to seeing other people affected by HIV, regaining physical health, returning to productive activities and receiving emotional support from health service providers. Overcoming internalized feelings of shame facilitated disclosure of HIV status, helped to sustain treatment, and stimulated VCT uptake. However "blaming" stigma - where people living with HIV were considered responsible for acquiring a "moral disease" - persisted in the community and anticipating it was a key barrier to disclosure and VCT uptake. Attributing HIV symptoms to witchcraft seemed an effective mechanism to transfer "blame" from the family unit to an external force but could lead to treatment interruption.

**Conclusion:**

As long as an HIV diagnosis continues to have moral connotations, a de-stigmatisation of HIV paralleling that occurring with diseases like cancer is unlikely to occur. Maximizing synergies between HIV treatment and prevention requires an enabling environment for HIV status disclosure, treatment continuation, and safer sexual behaviours. Local leaders should be informed and sensitised and communities mobilised to address the blame-dimension of HIV stigma.

## Background

Historically, sexually transmitted infections like syphilis have been feared, considered as "invaders of the community", attributed to a "spoilt identity" [1] and perceived as a deserved punishment. Other infectious diseases like leprosy have also been feared [2], especially if they led to progressive physical deterioration [3,4] or as in the case of cholera, if related to sudden death and accompanied by "un-dignifying" symptoms [5]. Untreatable non-infectious diseases have also been heavily stigmatised due to their association with an unavoidable death. However, as the case of cancer shows, stigmatised diseases previously seen as a "death sentence" tend to be progressively perceived as "any another disease" and to "normalise", once effective therapy is made available [5].

Following this logic, the provision of antiretroviral therapy (ART) in resource-poor settings was expected to mitigate stigma by turning AIDS into a manageable condition, subsequently leading to improved rates of disclosure and HIV testing [6,7], and ultimately to safer sexual behaviours. Experiences in resource-poor settings have indeed demonstrated the potential of treatment availability to reduce stigma and strengthen HIV prevention [8-10]. However, stigma continues to be a crucial barrier to HIV status disclosure [11-13], treatment adherence [13-15] and uptake of voluntary counselling and testing (VCT) [16-22]. This is of particular relevance in the context of generalised epidemics in sub-Saharan Africa where 22 million people live with HIV/AIDS [23], most of whom are unaware of their own and their partner's HIV status [24,25], and where between 45% and 75% of married HIV-positive individuals have HIV-negative spouses [26,27].

While theoretical approaches to understanding HIV-related stigma have received substantial attention in the literature [28-31], few empirical studies have explored the impact of ART availability on different forms of stigma in resource-poor settings. As part of a broader study exploring attendance rates at an ART clinic in a semi-rural ward of northern Tanzania [32,33], this study investigated the impact of ART provision on perceptions of HIV and its implications for prevention.

### Theoretical framework

Early work considered stigma as a "discrediting attribute" enacted through interpersonal interactions which spoiled and devaluated an individual's social identity [1,34]. However, "labelling" interpretations of stigma as an individual's static characteristic or "undesirable difference" have led to interventions focused at the individual level which overlook social and structural determinants such as the role of families, communities, national polices and the economic context [31,35]. Broader frameworks that question restrictive interpretations of Goffman's work and incorporate Foucault's reflections on culture, power, and the "social production of difference" have been proposed. These consider stigma as a social process used by individuals, communities and the state to produce and reproduce relations of power and control [28]. Stigma thus becomes part of a complex "struggle for power" and can serve as a form of "social psychological policing" [36] aimed at perpetuating the advantage of some groups in favour of others, legitimizing hierarchies and inequalities of class, race, gender and sexuality [28].

Drawing from the conceptual work of Deacon, we understand stigma as a culturally constructed and constantly changing social process, through which people project blame onto "out groups" in an emotional response to perceived danger [30]. This process, originating in the desire to avoid diseases [37], creates boundaries between the "normal" and those posing moral and health threats to the majority, and *in certain circumstances*, may be used as an instrument to maintain the "social order" [28,30,38,39]. Using the recent typology developed by Phelan and colleagues, we consider that stigma is based on both "disease avoidance" (*keeping them away*) and "norm enforcement" (*keeping the rest in*) [40].

Based on distinctions made between the *"emic" *or insider's perspective and the *"etic" *view of outsiders [41], we differentiate *enacted *stigma or discrimination from *self-stigma*, defined as the internalised feelings of shame resulting from accepting others' judgments over one's identity [1,30] which often lead to constricted social networks [13], low self-esteem, and hopelessness [29,42]. *Anticipated *stigma will be understood as the reactions people expect from others if it were to become known that they were living with HIV [30]. Stigma can be projected onto those closely related to people living with HIV/AIDS (PLHA) such as family members who may share the shame and blame attached to those infected (*secondary *stigma) [29,43] and such layered disadvantage can lead to *multiplying stigma *[44].

As for the sources of stigma, we differentiate between 1) *burden*-related stigma, derived from the inability of individuals to conduct productive activities and look after themselves, 2) *blame*-related stigma, resulting from the association of PLHA with negatively-defined behaviours and 3) stigma derived from the *fear *of being infected by HIV through casual or sexual contact [30,45].

For the purpose of this study we define "HIV normalisation" as a process closely linked to the existence of effective treatment [46] whereby persons living with HIV feel progressively re-integrated into productive and social life.

### Study setting

Data collection took place in Kisesa ward, in Magu district, where a community-based cohort study, operating in this community since 1994, is currently monitoring the implementation of the national ART programme. Kisesa has a population of approximately 29,000 inhabitants and consists of five dispersed villages and a trade centre along the main road from Mwanza city to the Kenyan border. Farming and petty business are the main economic activities and the income per capita is below 120 USD per year. Traditional religions are followed by 23% of the population, while 74% are Christian and 3% are Muslim. Over 37% of deaths among individuals above 15 years of age are attributed to HIV/AIDS in this community and HIV prevalence was estimated at 7.4% in 2006/7.

Since 2003, VCT services have been available from temporary village-based facilities provided during HIV surveillance rounds which are conducted every two-three years. VCT services have additionally been available from a permanent clinic located in the trade centre since 2005. Referrals to the ART clinic, located in Mwanza city, about half an hour's drive away, have been facilitated by these VCT services since the beginning of the national HIV treatment programme in 2005. Eligibility criteria for treatment initiation include disclosure of HIV status to at least one "treatment supporter" and being registered with a community-based organisation (CBO) providing home-based care services (HBC) in the ward.

## Methods

Qualitative tools were used including semi-structured interviews with 42 clients and 11 HIV service providers from the ART Clinic, the VCT clinic and the CBO providing HBC services in the ward (Tables 1 and 2). Four participatory activities were conducted with members of a post-test support group (Table 3) using visual tools to identify and rank barriers to clinic attendance. A sampling frame was constructed that consisted of all Kisesa residents who had registered at the ART clinic. Participants were then purposively selected to broadly reflect the composition of the study population in terms of sex, area of residence, ART status and adherence to clinic appointments, as shown in table 1.

**Table 1:** Characteristics of the sampling frame and ART clinic clients undertaking semi-structured interviews

		*In sampling frame*	*Interviewed*	
**Variable**	**Category**	***N***	***n (%)***	

**Total**		**66**	**42**	**(64)**

Sex	Male	20	14	(70)
	Female	46	28	(61)

Age	15-24	4	2	(50)
	25-34	29	21	(72)
	35-44	22	16	(73)
	45+	11	3	(27)

Area of residence	Remote rural	16	11	(69)
	Roadside	50	31	(62)

ART status	On ART (no interruptions)	33	21	(64)
	On ART (at least 1 interruption > 1 month)	4	4	(100)
	Not yet started ART	29	17	(59)

Number of missed appointments (pre- and post-ART initiation)	0	34	21	(62)
	1	13	10	(77)
	2	10	6	(60)
	>2	9	5	(56)

Period referred from VCT	Mar 05 -Sep 05	28	17	(61)
	Sep 05 -Feb 06	10	7	(70)
	Mar 06 -Sep 06	28	18	(64)

**Table 2:** Characteristics of service providers undertaking semi-structured interviews

Type of service provider	*n*
VCT Counsellors	2

Registration nurses	2

Adherence counsellors	1

Tracing nurse	1

Home-based care providers	3

ART Doctor	2

**Sex**	

Females	9

Males	2

**Table 3:** Characteristics of participants in group activities

Group 1	Men, road-side areas (n = 11)
Group 2	Men, remote areas (n = 6)

Group 3	Females, road-side areas (n = 18)

Group 4	Females, remote areas (n = 11)

The VCT counsellors and HBC providers delivered the interview invitations to their clients during counselling sessions or home visits, while the invitation to participate in the group discussion was made during a scheduled meeting of the post-test club. All invited individuals were informed about the overall aims of the study and its methods.

Prior to starting the interviews, participants were informed that they could stop at any time and choose not to answer any of the questions. In order to minimise distress, group discussions focused on the expected experiences of a "hypothetical community member" while accessing HIV services, rather than the specific experiences of the participants. Consent was obtained and tape recorded at the beginning of each interview and was verbally obtained from each person participating in the group discussions. (see additional file 1).

Participants were not offered any economic incentive beyond a payment of 2000 Tanzanian shillings (equivalent to ~2 USD) to compensate for their time and transport costs. Interviewers and group discussion facilitators received training that was specific to the sensitive nature of the topic, and which also covered ethical issues such as confidentiality. To ensure anonymity, codes were used for identifying participants on all tapes and transcripts. At the end each activity participants were informed about the availability of supportive counselling services provided at the VCT clinic and were given detailed information about HIV and how to access the ART clinic. Ethical approval was obtained from both the London School of Hygiene and Tropical Medicine and the Tanzanian Medical Research Coordinating Committee.

All the interviews were recorded after permission was granted, and the data were subsequently transcribed and translated into English. For the group activities, detailed notes were taken by one of the researchers. Data were analyzed with the qualitative software package NVIVO-7 using a social ecological framework as an initial coding guide to identify manifestations of HIV-related stigma at interpersonal, community, and wider social levels [47-49]. This was followed by detailed analysis of the main *types *(enacted, internalised, anticipated, secondary) and *sources *of stigma (fear, blame, burden) conceptualised in the existing literature [30]. Respondents were classified by demographic variables which allowed us to explore patterns by gender, residence and age. To maximise rigour, the interpretations were validated by a second researcher who independently coded the data and emerging hypotheses were tested by actively seeking counter examples or "dissenting voices".

## Results

### Decreased internalized stigma

A process of reduced self-stigma was frequently experienced by ART users participating in this study. Reports about "feeling like an ordinary person" were often expressed by both men and women and especially by residents of the trade centre.

*"I just felt good because this disease has now become like an ordinary disease" *(female, roadside area)

The actual or expected positive outcomes derived from being on ART played a major role in helping individuals to overcome internalised feelings of shame (*"aibu"*).

*"... you will stop being embarrassed. Do you want me to die? So you stop feeling shame. You do away with shyness" *(group activity, females from roadside areas)

The increased public profile of PLHA in the media and interaction with other patients at the ART clinic contributed to greater awareness about how widespread the disease was and to a comforting realisation of *"not being the only one"*.

*"I see that just as usual...because I am not the only one...it is not at one family. Now it is the whole of Tanzania" *(female, roadside area)

*"It's not me alone. There are so many people too. In fact they are many. Others are even older that I am. Ee, it's many, you see, so I continued encouraging myself" *(female, roadside area)

In addition to acknowledging the significant numbers sharing the experience of HIV, participants highlighted their growing awareness that AIDS could affect a wide range of community members, including people deemed "innocent" and "respectable", which presumably indicated those who did not exhibit characteristics or behaviour usually associated with the shame ascribed to the disease.

*"Later I perceived it to be just normal. Normal...because this is not a disease that's perhaps to me alone. You see it advertised on newspapers, it takes even the members of parliament and we see it" *(male, roadside area)

*"I felt just better because at the beginning we hadn't seen such a number of children also having the virus while they don't even know about sexual matters or anything" *(female, roadside area)

Respondents also identified emotional support from health service providers and other ART users as playing a significant role in easing anxiety and treating HIV as any other manageable disorder found in the community.

*"Many patients come here stigmatizing themselves psychologically. We explain and educate them... after they see that crowd of people they agree and even apologize: 'I thought it was only me, but this problem is just normal and we are many"' *(service provider, ART clinic)

*"The doctor told me: 'you have been infected with the disease'. After I received good instructions it encouraged me. I stopped worrying. It's when I got courage that I should perceive it to be just a normal thing" *(female, roadside area)

*"We reassure the patients that there is no problem: 'there are so many people like you. This service is like the service on diabetes, heart patients'..." *(service provider, ART clinic)

This process of "normalisation" had three demonstrable positive outcomes: (1) facilitating HIV status disclosure, (2) stimulating VCT uptake, and (3) helping ART users cope with enacted stigma and sustain treatment.

Some study participants mentioned that they had disclosed to family members *only after *having initiated therapy and a few described how the "normalisation" of HIV had facilitated the process of disclosure and their acceptance within their own families.

*"I felt that I should involve my relatives. I told my brother, my father. I also told my sister-in-law. She told me that I should just be calm, 'you just get treatment, this has become just like fever, you shouldn't look back"' *(female, roadside area)

*"...after returning there (ART clinic) I started involving her (wife). We would go there often with her and other patients and I would show her: 'all these are suffering from the same disease'... gradually she started perceiving it to be just normal" *(male, roadside area)

*"He (brother) just gave me hope because I am not the only one. There are just many people who are in that condition and they are continuing to live. There is hope because being affected is not the end of life" *(male, roadside area)

*"The catastrophe is now for everybody, I can't keep it to myself, I have to tell my relatives... They just said that this disease is now a disease for all the people" *(female, roadside area)

For some however, the fear of admitting their status persisted and reflected the very real negative consequences that could ensue:

*"I told him and he said: if you are in that condition then we will have to separate" *(female, remote rural area)

The positive effects of initiating therapy and the reduction of internalized stigma led some ART users to successfully advise others to undergo HIV testing and initiate treatment.

*"I had to tell him/her because you know there is no problem. S/he said I will go to check too" *(male, remote rural area)

*"A neighbour told me that he had been using them for a long period. He took me there...I started using these drugs and until now I am continuing using them" *(female, roadside area)

Finally, many clients reported how the reduction of internalized stigma helped them cope with community-level stigma and sustain treatment.

*"You just ignore them. You know you are sick. Why should you listen to him/her? You have only to ignore him/her. What I care is only to go to get the drugs. Let him/her talk...definitely they will say something but I will just continue taking the drugs" *(female, roadside area)

### Persistence of enacted and anticipated stigma

In spite of the reduction of internalised stigma and the trend towards a "normalisation" of HIV, participants sometimes referred to having been excluded, mocked, feared and blamed by members of their community. AIDS was still perceived as an avoidable "moral disease" associated with unacceptable sexual behaviour and PLHA were consequently judged to be personally responsible for contracting HIV. While burden-related stigma as well as the fear of HIV transmission through casual contact had decreased, blame-related stigma persisted in spite of the generalised epidemic and the availability of ART.

Only a few cases reported experiencing severe stigma at home compared to those reporting discrimination from community members. However, when stigma was experienced within the family, its consequences could be harder to overcome and even result in treatment interruption.

Participants often referred to a wide range of attitudes towards PLHA in this community, with this heterogeneity making it difficult for them to predict the stigma they might face in the event of disclosure. The unpredictable nature of these responses contributed to persistently high levels of anticipated stigma, and prevented some participants from disclosing their HIV status.

*"You find some pointing a finger at you: 'that one has AIDS', they are stigmatizing us, but others see it just as malaria fever" *(female, roadside area)

*"I didn't tell anyone...because I felt that if I will tell someone, most of the people are not good, there are some who will keep on talking things about you. There are others who may see that as something strange, another may feel sad for someone. You can't know" *(male, remote rural area)

Anticipated stigma contributed to explaining the resistance of some family members to accept the HIV status of a relative. Witchcraft seemed to be a more sociably acceptable explanation for the disease and a few people believed that HIV could be cured through prayers. Family-level "denial" could heavily influence individuals' health-seeking behaviour, preventing HIV testing and even leading to attrition from the ART program.

*"...that family isolated him/her. They used to attend him/her thinking that s/he was bewitched but since then I have to attend him/her myself" *(service provider, HBC organisation)

*"My husband suffered and his relatives took him to traditional medicines...I begged him till he agreed (to test), his relatives were refusing"*(female, roadside area).

*"He/she comes to tell you again: 'I was told at home...that I should not go to hospital but I should go to church"' *(service provider, HBC organisation)

Condom use was stigmatised by association with illicit sexual activity, providing evidence of the widespread perception that HIV was a result of degrading behaviour.

*"If you tell a woman about condom. she will think that you have regarded her to be garbage" *(male, roadside area)

Finally, it is worth highlighting occasional reports that pointed to ART availability as a factor that could potentially feed into existing community-level stigmatisation of PLHA as a consequence of their regained health and perceived increase in sexual activity.

*"I just used to hear that they have brought medicines for increasing life, now that fear: why do they bring us those medicines? People taking them will continue to do evils and spread it. But I didn't know that I too was infected" *(male, roadside area)

## Discussion

**Figure 1:**
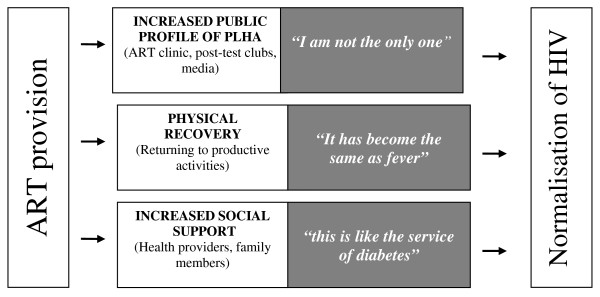
**Impact of ART provision on HIV "normalisation"**.

Our data suggest that a decrease in self-stigma is experienced by ART users reflecting a certain "normalisation" of the disease. By seeing lots of other people affected during visits to the clinic, as well as regaining physical health, engaging in productive activities and receiving emotional support from health service providers and family members, ART users tend to progressively perceive HIV as "an ordinary disease" which affects many other "normal people". (Figure 1)

As initially hypothesized, the provision of HIV treatment has thus had a powerful impact on the stigma felt by many ART users. This can facilitate HIV status disclosure, stimulate VCT uptake and lead to safer sexual behaviours [8,9]. Overcoming internalised stigma also helps to cope with enacted stigma and sustain treatment, which in turn leads to lower infectiousness [50,51] further maximising potential synergies between HIV treatment and prevention [52].

However, in spite of reduced internalised stigma reported by most ART users, attitudes towards PLHA in the community were very heterogeneous and discrimination prevailed. The "blame" discourse behind *enacted *stigma was only marginally affected by ART availability and the deeply rooted association of HIV/AIDS with *shameful and avoidable behaviour *persisted. Research conducted in the same study setting points to the emergence of new sources of stigma associated with the perception that people on antiretroviral treatment could contribute to the ongoing spread of the disease. [53]

Our findings suggest that ambivalent and heterogeneous attitudes towards PLHA result in high levels of *anticipated stigma*, which may prevent HIV status disclosure. Associations between anticipated stigma and non-disclosure [54] help to explain why barriers to disclosure persist in spite of antiretroviral availability [11-13]. The lack of a correlation between being on ART and disclosing HIV status identified in other studies [55] is of particular relevance considering the potential increase of sexual activity among ART users as their health improves [56,57] and the strong associations found between non-disclosure, higher risk behaviours [58], lower adherence [59,60], and treatment failure [61].

Denial of HIV status also appeared to be linked to anticipated stigma and could lead to treatment interruption. The limited impact of ART provision on the "blame" dimension of stigma, seemed to make healing alternatives such as *"being prayed for" *and causal explanations such as *"being bewitched" *more acceptable. The latter might particularly affect family members wishing to switch the burden of "shame and blame" away from the family by attributing the disease to an uncontrollable force like the deliberate action of witches rather than to the "immoral and avoidable behaviour" of a family member. Similarly, not testing for HIV would be an effective strategy to perpetuate ambiguity over the real causes of the syndrome.

Our findings suggest that the "moral exploitation" of HIV as a tool used by some groups to distance them from perceived risk and police the behaviours of others might prevent the benefits of ART from being maximised. The potential positive effects of ART provision on status disclosure, viral load reduction, and VCT uptake risk being eroded by persistent blaming attitudes that lead to high levels of anticipated stigma and contribute to HIV denial. Stigma projected onto condoms and recent moves towards the criminalisation of sexual and vertical transmission of HIV in a number of sub-Saharan African countries further undermine the potential of ART provision to contribute to prevention efforts [62].

While health service providers can effectively contribute to tackling internalised and enacted stigma [35], anti-stigma interventions should target PLHA as well as their families if the full public health benefits of ART roll-out are to be realised. Local leaders should be informed and sensitised and communities mobilised to address the blame-dimension of HIV stigma. Sharp increases in VCT uptake took place in Tanzania, after government personalities tested for HIV in July 2007 [63]. This demonstrates that opinion leaders can effectively influence values and contribute to a reduction of stigma by emphasizing that HIV is not a "deserved punishment" but a "normal disease" that can affect anyone.

The underlying causes of stigma are similar across a wide diversity of countries and continents [29]. However, caution is needed before generalizing our results. The small sample size that characterises qualitative studies poses certain limits to the extrapolation of our findings, particularly when we take into account that individuals accessing the ART clinic during the two first years of availability might be those who were most motivated and in the most favourable circumstances. The small study sample also limits our ability to identify patterns by sex and age. Furthermore, positive experiences might have been over-reported due to "courtesy bias". Finally, while the attribution of HIV disease to witchcraft is common in rural Africa [64-66] and a tendency to "*hide behind witchcraft" *has been identified elsewhere [67], AIDS symptoms may also be attributed to ancestors who "punish an individual for having transgressed". As even traditional beliefs in ancestral powers may attribute a certain level of blame to affected individuals, HIV education needs to counter stigma whatever its source [68].

This study was limited to clients' and HIV service providers' perceptions and additional research is required to explore the social consequences of ART scale-up from a wider community perspective. Perceived and actual changes in the sexual behaviours of PLHA need particular attention. In the long run, if the widespread use of antiretroviral drugs has a significant impact on viral load levels and on users' infectiousness, this may also become apparent in the community and have a substantial impact on stigma.

## Conclusion

As long as a HIV diagnosis continues to carry moral connotations, widespread de-stigmatisation of HIV mirroring that of diseases like cancer is unlikely to occur. The potential positive effects of ART provision on status disclosure, viral load reduction, and VCT uptake are at risk of being eroded by persistent blaming attitudes. Key community stakeholders should be identified, trained and sensitised to tackle the blame-dimension of HIV stigma and create an enabling environment for HIV disclosure, treatment continuation, and safer sexual behaviours.

## Competing interests

The authors declare that they have no competing interests.

## Authors' contributions

MR wrote the first draft of the paper, co-designed the study, conducted analysis and trained field-workers. AW co-designed the study, conducted analysis and contributed to drafting. JB provided technical advice, trained field workers and assisted in drafting the paper. BN and DM facilitated group activities and conducted interviews. BZ, technical advisor for the whole cohort study, provided overall advice and contributed to drafting the paper. MU, director of the cohort study, facilitated the coordination of field-work and contributed to drafting the paper. All authors read and approved the final manuscript.

## Pre-publication history

The pre-publication history for this paper can be accessed here:

http://www.biomedcentral.com/1472-698X/9/22/prepub

## Supplementary Material

Additional file 1 **Informed consent forms**. The full text of the informed consent forms used to recruit study participants is provided. Click here for file
