# Supplementary material for: "Just like fever": a qualitative study on the impact of antiretroviral provision on the normalisation of HIV in rural Tanzania and its implications for prevention
Source: BMC Int Health Hum Rights. 2009 Sep 9;9:22. doi: 10.1186/1472-698X-9-22 (PMC2759900; doi:10.1186/1472-698X-9-22)
Supplement: Additional file 1 — Informed consent forms. The full text of the informed consent forms used to recruit study participants is provided. [file 1472-698X-9-22-S1.doc]

**Informed Consent Sheet**

Interviews with individuals referred to Care and Treatment Clinics

Thank the participant for his/her time and introduce yourself. Remind the participant of the following:

- The Tanzanian government is making HIV medication, including antiretroviral therapy (also known as ART) available to HIV-infected people through regional hospitals. The Bugando Medical Centre and ST Hospital are the closest ART programme sites to Kisesa.
- NIMR is implementing a project to help Kisesa cohort communities become among the first to gain access to the new ART programme. This includes putting in place a referral and follow-up system and adapting it as the need arises, in order to make it as effective as possible.

Explain to participants that:

- NIMR is carrying out a study in order to obtain information about the ART programme, and in particular, the referral process which patients go through in order to access ART. The study is also documenting the challenges that patients have faced during the referral process, in order to explore ways in which these challenges may be overcome.
- It is important for us to hear about the different experiences, concerns, and suggestions of people who have already attended appointments at the Care and Treatment Clinics (CTCs), either at Bugando and/or ST.
- We are interested in finding out about what you know about HIV and the requirements for initiating ART.
- We are also interested in learning about your experiences of the referral process and any barriers that you may have found in relation to attending the CTC. The reason we want to learn about the difficulties you might have experienced is so that NIMR can improve the referral process, and facilitate access to ART for people in need of treatment. While we will do our best, we cannot guarantee that we will be able to cover all the needs that are identified by study participants.

If you have any questions about the information that I have given you, or about any aspect of the study, then please feel free to ask me and I’ll try to clarify the information for you.

Do you have any questions?

If you have additional questions regarding the study after the discussion, you can speak with me or contact the VCT counsellors, the fieldworker in your village that can then get in touch with Raymond Nsigaye - Clinical Officer at NIMR (Phone: XXX), or Ms. Louise Kaswamila, field supervisor at TUMAINI (Phone: XXX).

This interview will take about 1 hour. It will be kept completely confidential within the study. Codes will be used for identification so we will not record your name anywhere. All the professionals involved have been trained and are fully committed to keep this information confidential. We would, however, like to tape record this interview to help with our documentation. Only researchers at NIMR will hear this tape. Do I have permission to record our conversation? *– turn on tape recorder if permission given*

*(Alternatively the interviewer should read the attached “Consent Form” to the interviewee and sign it as a witness).*

In this interview, I am going to ask you for some personal information about your experiences along the referral process and at the CTC. Some of the questions may bring up issues or emotions that are upsetting or difficult for you, but I will try to offer counselling and answer your questions throughout our conversation. You do not have to answer any questions that you do not want to, and you can ask to stop the interview at any time.

Do you agree to continue with this interview?

**Consent Form**

I would like to talk to you about your experiences participating in the ART programme and the referral process.

This interview will take about 1 hour. Though I will be taking notes, all the information will be kept completely confidential. We will be using codes to identify you, so your name will not be recorded anywhere. All the professionals involved in the project have been trained and are fully committed to keep information confidential.

In this interview, I am going to ask you for some personal information about your experiences during the referral process, and at the CTC. Some of the questions may bring up issues or emotions that are upsetting or difficult for you, but I will try to offer counselling and answer your questions throughout our conversation.

You do not have to answer any questions that you do not want to, and you can ask to stop the interview at any time.

Do you have any question about what I have just explained?

Do you agree to participate in this interview?

_____________________ _______________

Signature of participant Date

_____________________ _______________

Signature of Witness (Interviewer) Date

**Informed Consent Sheet**

Group Activity with members of the Kisesa post-test club

Thank the participants for their time. Introduce yourself and the note taker. Ask each participant to introduce themselves to the whole group in any way they wish.

Remind participants that:

- The Tanzanian government is making HIV medication available to HIV-infected people through regional hospitals. The Bugando Medical Centre and ST Hospital are the closest ART programme sites to Kisesa.
- NIMR is implementing a project to help Kisesa cohort communities become among the first to gain access to the new ART programme. This includes putting in place a referral and follow-up system, and adapting it in order to cover the needs identified.

Give a brief overview of the study, including the following:

- NIMR is carrying out a study in order to obtain information on different aspects of the ART programme, and in particular, on the referral process between Kisesa and the CTC. The study is also documenting any challenges which have been experienced, in order to explore ways in which these areas could be improved.
- It is important for us to hear about the general experiences, concerns, and suggestions of people who have already attended appointments at the CTCs of Bugando and/or ST.
- We are interested in learning about your common attitudes and experiences of the referral process and the barriers to firsts and repeated attendance to CTC. We are also interested in knowing what is understood about HIV disease progression and the requirements for initiation of ART.
- The reason we want to learn about the difficulties that you might have experienced in attending CTC is so that NIMR can try to make the process as easy as possible. While we will do our best, we cannot guarantee that we will be able to cover all the needs that are identified in the study.
- The purpose of this participatory group activity will be to *exchange* information. We will ask you to share knowledge, experiences and perceptions on ART in general and the referral process and pre-ART appointments in particular. Then we will clarify any questions or misconceptions that might arise. We will also explore which barriers to accessing the ART are the most important to you, as well as trying to identify potential measures that could be put in place to overcome them.
- We will be discussing as a *group,* learning about the programme in general and identifying common problems, and ideas for possible solutions to these problems. Although the discussion will be kept confidential by the researchers, no one needs to share any personal experiences or discuss sensitive topics.
- Codes will be used for identification so we will not record your names anywhere. Notes on the discussion will be taken, however, and these will be taken back to the NIMR office in Mwanza to be shared with other researchers who are working on the monitoring of the ART referral programme. All the professionals involved in this project have been trained and are fully committed to keep all the information confidential.
- It is possible that the discussion might bring up difficult and upsetting issues. Any participant is free to leave at any time or to not participate in any part of the activity.
- If you have any questions about the information that I have provided, or about any other aspect of the study, then please feel free to ask us now.

If you have additional questions regarding the study after the discussion, you can speak with me or contact the VCT counsellors, the fieldworker in your village that can then get in touch with Raymond Nsigaye - Clinical Officer at NIMR (Phone: XXX), or Ms. Louise Kaswamila, field supervisor at TUMAINI (Phone: XXX).

- Do all of you agree to continue with this activity?

(Note: Prior to the focus group activity, the member of TUMAINI staff in charge of recruiting participants will have already shared general information about the project in one of the usual post-test (PLHA) club meetings. She will by then explain that the group activity will be a participatory exercise based on information exchange and conducted in single sex groups of 8-12 participants each).

**Informed Consent Sheet**

Interviews with service providers

**Information Sheet** (to be kept by the interviewee)

NIMR is currently conducting a research project with the objective of investigating the uptake and impact of antiretroviral therapy at the community level in Kisesa.

As part of this research project, NIMR is facilitating access to HIV care and treatment services for HIV-positive people in the Kisesa community, through collaboration with Bugando Medical Centre and Tumaini Home Based Care. This includes putting in place a referral system for HIV-positive patients, and adapting it as needs be, in order to improve its efficiency.

In this specific sub-study, we are interested in documenting the changes that have been made to the referral system since its initiation, and identifying ongoing barriers or facilitating factors to patients accessing ART, from the perspective of key healthcare workers. We are conducting in-depth interviews with people living with HIV/AIDS and also conducting semi-structured interviews with health-care professionals involved in the provision of referral services and HIV care and treatment.

The aim of the interviews with the health care professionals is to identify on-going barriers or facilitating factors to patients accessing ART, from the their perspective, as well as discussing potential solutions to barriers that may be impeding the efficient functioning of the referral system. Due to your expertise in this field, we would like to learn about your experiences in this area. We are interested to know about any ideas that you may have about future improvements to the referral system, so that NIMR can try to make the process as efficient as possible for patients in the future.

While we will do our best, we cannot guarantee that we will be able to implement all the suggestions made during the study.

If you have any questions about the information that I’ve given you, or about the study, please feel free to ask, and I’ll do my best to answer.

If you have further questions regarding the study after this conversation, you can contact Mark Urassa, the head of the TAZAMA ART research programme, based at NIMR (Phone: XXX)

**Consent Form**

Thank you for taking the time to meet with me today. I would like to talk to you about your experiences participating in the ART programme and the referral process.

This interview will take about 1 hour. It will be kept completely confidential within the study. We will be using codes to identify participants so your name will only be recorded in the present form which will be kept in a file only accessible to project staff. All the professionals involved in the project have been trained and are fully committed to keep information confidential. The transcriptions and translations will be carried out by an internal member of the research team.

We will be producing a report in order to incorporate your views and disseminate the findings of this research. We will attribute certain statements to “a Home Based Care Provider”, or “a Health Worker”. By using such general categories we will ensure that you can not be identified as the respondent.

We would like to tape record this interview to help with our documentation. Only researchers at NIMR will hear this tape.

You do not have to answer any questions that you do not want to, and you can ask to stop the interview at any time.

Do you have any question about what I have just explained?

Do you agree to participate in this interview?

Do I have permission to record our conversation?

________________ _________________

Signature of Interviewee Date
